# Genome-Wide Analysis of the Apple CBL Family Reveals That Mdcbl10.1 Functions Positively in Modulating Apple Salt Tolerance

**DOI:** 10.3390/ijms222212430

**Published:** 2021-11-18

**Authors:** Peihong Chen, Jie Yang, Quanlin Mei, Huayu Liu, Yunpeng Cheng, Fengwang Ma, Ke Mao

**Affiliations:** State Key Laboratory of Crop Stress Biology for Arid Areas/Shaanxi Key Laboratory of Apple, College of Horticulture, Northwest A & F University, Xianyang 712100, China; cph0219@163.com (P.C.); yangjie320@163.com (J.Y.); mqlyes@163.com (Q.M.); huayuliu93@163.com (H.L.); cyp2020055284@163.com (Y.C.)

**Keywords:** *Malus domestica*, calcium, calcineurin B-like proteins, Na^+^ accumulation, salt tolerance

## Abstract

Abiotic stresses are increasingly harmful to crop yield and quality. Calcium and its signaling pathway play an important role in modulating plant stress tolerance. As specific Ca^2+^ sensors, calcineurin B-like (CBL) proteins play vital roles in plant stress response and calcium signaling. The CBL family has been identified in many plant species; however, the characterization of the CBL family and the functional study of apple MdCBL proteins in salt response have yet to be conducted in apple. In this study, 11 *MdCBL* genes were identified from the apple genome. The coding sequences of these *MdCBL* genes were cloned, and the gene structure and conserved motifs were analyzed in detail. The phylogenetic analysis indicated that these MdCBL proteins could be divided into four groups. The functional identification in Na^+^-sensitive yeast mutant showed that the overexpression of seven *MdCBL* genes could confer enhanced salt stress resistance in transgenic yeast. The function of *MdCBL10.1* in regulating salt tolerance was also verified in cisgenic apple calli and apple plants. These results provided valuable insights for future research examining the function and mechanism of CBL proteins in regulating apple salt tolerance.

## 1. Introduction

Plants are inevitably exposed to a variety of adverse environmental conditions due to their sessile lifestyle. They have evolved a series of signal transduction mechanisms to fine-regulate the body’s adaptation to the environment. Calcium ion (Ca^2+^) is a nutrient element crucial for plant growth and development. It also acts as the ubiquitous intracellular secondary messenger initiating the Ca^2+^ signaling pathway, which is a fine regulatory mechanism responsible for the acquisition, perception, transformation, transmission, and decryption of external stimuli [1]. Ca^2+^ signals are an important regulator of growth, development, and biotic and abiotic stresses in plants. Refs. [2,3,4,5]. Under stress conditions, Ca^2+^ signals induced by the stimulus are first perceived, decoded, and transmitted by Ca^2+^ sensors [6].

Based on the protein structural characteristics, Ca^2+^ sensors are divided into two types of sensors: sensor relays and sensor responders [7,8]. Sensor relays include calmodulin (CaM)-like proteins (CMLs) and calcineurin B-like (CBL) proteins, which do not have kinase activity [8,9]. They specifically target downstream proteins to transfer the perceived calcium signals. Sensor responder proteins, such as CaMs and Ca^2+^-dependent protein kinases (CDPKs), have all the functions of Ca^2+^ sensor relay proteins as well as the kinase activity [8,10,11]. As a result, CaMs, CMLs, CDPKs, and CBLs constitute sensors in the Ca^2+^ signal transduction pathway [12]. CaM is a ubiquitous conserved Ca^2+^-binding protein found in both animals and plants. The CML family was identified as encoding proteins that contain the CaM-like EF-hand structures and share at least 16% homology with CaM in amino acid residues [13,14,15,16]. CDPK comprises a kinase domain and a CaM-like domain (four EF-hands) in a single protein; thus, it acts as not only a Ca^2+^ sensor but also an effector [17,18,19]. The CBL family belongs to a unique group of calcium sensors in plants. Ca^2+^ can bind to the elongation factor (EF) hand domains of the CBL proteins, which changes their phosphorylation status. The change in the phosphorylation status of Ca^2+^ sensors activates several protein kinases, which sometimes lead to a protein phosphorylation cascade [6,20,21].

Decades of research have revealed that the CBL family proteins play important roles in plant stress response and resistance regulation. For example, CBL1 functions under drought, high salt, and hyperosmotic stresses in plants [22,23]. CBL2 and CBL3 regulate ion homeostasis across the vacuolar membranes under salt stress. Studies also proved that CBLs were involved in K^+^ regulation, which indirectly regulated the Na^+^ homeostasis. For example, AtCBL1/AtCBL9 regulates plant K^+^ homeostasis and salt tolerance by regulating the K^+^ channel AKT1 and K^+^ transporter HAK5 [3,24,25], while CBL4 modulates the activity of the plasma membrane K^+^ channel AKT2 [26]. The involvement of CBL in regulating plant salt stress response has been widely reported in a CIPK-dependent manner. The first identified CBL-CIPK pathway was the salt overly sensitive (SOS) pathway in *Arabidopsis*. Under salt stress, SOS3 (CBL4) interacts with SOS2, and the SOS2–SOS3 complex activates the transport properties of the cell membrane-located SOS1 to promote the Na^+^ efflux, thereby enhancing plant salt tolerance [27,28]. Other studies also found that the SOS3 homolog SOS3-like calcium-binding protein8 (SCABP8)/CBL10 interacted with SOS2 and enhanced plant salt tolerance by activating SOS1 [28,29,30,31]. Because of the crucial roles of CBL family proteins in plant growth and stress response, this family has been identified at the genome-wide level in many plant species [1,8,32,33,34]. For example, ten CBLs are present in *Arabidopsis* and rice [35], eight in pineapples [32], nine in peppers [10], and five in eggplant [36]. However, no detailed identification and characterization of the apple CBL family have been reported till now.

Apple (*Malus domestica*) is one of the most widely grown and economically valuable fruit crops globally. Abiotic stresses, such as high salinity, severely restrict its global yield and quality. Although CBL proteins play an important role in regulating plant salt tolerance, little is known regarding the function of apple CBL proteins in salt stress response. Genome-wide identification and gene cloning of CBL family genes were performed in apples in this study. The collinearity, phylogenetic relationship, gene structure, and conserved motifs of these MdCBLs were analyzed in detail. The functional identification in the Na^+^-sensitive yeast mutant showed that several *MdCBLs* played positive roles in modulating salt response. The function of MdCBL10.1 to enhance apple salt tolerance was verified in cisgenic apple calli and apple plants. These results provided valuable insights for subsequent research on the functions and regulatory mechanisms of MdCBLs in apples. 

## 2. Materials and Methods

### 2.1. Sequence Retrieval and Identification of Apple CBL Family Proteins

The apple proteome file (GDDH13_1-1_prot.fasta) was downloaded from the GDR database (Genome Database for Rosaceae; https://www.rosaceae.org/, accessed on 6 April 2021), and the protein sequences of 10 AtCBLs were downloaded from the TAIR (The Arabidopsis Information Resource) database (https://www.arabidopsis.org/, accessed on 6 April 2021). The HMM file EF-hand_7.hmm (PF13499.8) was downloaded from the Pfam database and used as a query to search the apple proteome. Phylogenetic analysis was subsequently performed with the protein sequences of the HMMER screening results and the 10 AtCBLs. Further, 11 MdCBL family proteins were identified from the phylogenetic tree (Figure S1). This result was also verified with the local BLASTp search using the 10 AtCBLs as queries, which was conducted with the BioEdit software (version 7.0.9.0).

### 2.2. Collinearity Analysis and Characterization of Apple CBL Family Genes

The GFF file (gene_models_20170612.gff3) that contained location data for apple CBL family genes was downloaded from the GDR database. The collinearity analysis between different apple chromosomes was performed with MCScanX software, and the results were visualized using TBtools software. The protein length, mass weight, p*I* (isoelectric point) values, and charge at pH 7.0 were determined with the DNAstar software (version 7.1.0). The best hits in *Arabidopsis* for MdCBL proteins were determined by the local BLASTp search.

### 2.3. Phylogenetic Relationships, Gene Structure, and Conserved Motif Analysis

Phylogenetic analyses were constructed with the MEGA-X software (version 10.0.5) using the neighbor-joining method (bootstrap method, 1000 replicates, Poisson model, pairwise deletion). The intron–exon schematic structures of *MdCBL* genes were drawn with the TB tools. The online MEME software (version 5.3.3; https://meme-suite.org/meme/tools/meme, accessed on 26 April 2021) was used to identify conserved motifs in the protein sequences of these MdCBLs. Detailed methods refer to previous studies [37,38].

### 2.4. Cis-Acting Elements in the Promoters of MdCBL Genes

The upstream regions (1500 bp) of MdCBL genes were obtained from the apple genome file (GDDH13_1-1_formatted.fasta) downloaded from the GDR database. Abiotic stress– or hormone response–related regulatory elements were identified using the PlantCARE software (http://bioinformatics.psb.ugent.be/webtools/plantcare/html/, accessed on 13 May 2021).

### 2.5. Vector Construction, Genetic Transformation, and Stress Treatment

For gene cloning of MdCBLs, total RNA was extracted from the leaves of “Golden Delicious” apple using a plant RNA isolation kit [Wolact, Vicband Life Sciences Company (HK) Limited] and reverse transcribed using a PrimeScript First Strand cDNA Synthesis Kit (Takara, Dalian, China). Gene-specific primers were designed based on the predicted coding sequences (CDSs) of these *MdCBLs* obtained from the GDR database.

For the transformation of yeast mutants, CDSs of *MdCBLs* were cloned into the pDR196 vector. The recombinant MdCBLs–pDR196 vector was transformed into the Na^+^-sensitive yeast mutant *Δena1-4* using the LiAc/ss carrier DNA/PEG method. After growth selection on selective medium (synthetic defined medium minus the appropriate amino acids) and PCR confirmation of transgene presence, three single colonies of each strain were selected for subsequent experiments. Detailed methods of yeast transformation and salt treatment referred to previous studies [39,40].

For the overexpression of the *MdCBL10.1* gene in the apple callus, the CDSs of *MdCBL10.1* were cloned into the pBI121 vector under the control of the 35S promoter. Detailed methods of genetic transformation and salt stress treatment on the apple calluses refer to previous studies [39,40]. 

To obtain the composite apple plants with *MdCBL10.1* expression whose expression increased or was interfered in roots, the CDSs and a 300-bp fragment of *MdCBL10.1* were cloned into the overexpression vector pCambia2300-GFP and the RNAi vector pK7GWIWG2D, respectively. Methods of *Agrobacterium rhizogenes* K599-mediated genetic transformation referred to previous studies [39,40]. For NaCl stress treatment, the plants in the treatment group were irrigated with 150 mM NaCl solution for 10 days at 5-day intervals. The same number of plants in the control group were irrigated with distilled water.

### 2.6. Measurement of Stress-Related Physiological Parameters

Relative electrolyte leakage (REL) and Na^+^ content were measured with a flame photometer (M410; Sherwood Scientific, Cambridge, UK) as described previously [39,40]. Malondialdehyde (MDA) content, H_2_O_2_ and O_2_^−^ content, and plant root activity were measured using Suzhou Comin Biotechnology test kits (Suzhou Comin Biotechnology Co., Ltd., Suzhou, China).

### 2.7. Statistical Analysis

IBM SPSS Statistics software (version 26; SPSS Inc., Chicago, IL, USA) was used for statistical analysis. Significant differences (*p* < 0.05) were determined using the Student *t* test or Tukey’s multiple range test.

## 3. Results

### 3.1. Identification, Characterization, and Gene Duplication of Apple CBL Family Genes

The HMM file (PF13499.8) was used as a query to search the apple proteome using the HMMER software (hmmsearch, version 3.1b2) so as to screen the CBL family proteins in apple. With default inclusion threshold, 230 protein sequences were obtained (Supplementary File S1). Then, the protein sequences of the ten *Arabidopsis* CBL family members were downloaded from the TAIR database. These ten AtCBL proteins, along with the 230 proteins in HMMER screening results, were used for phylogenetic analysis. Based on the phylogenetic tree (Figure S1), 11 proteins were finally identified as apple CBL family members and named based on their orthlogs in *Arabidopsis* (Table 1). The predicted protein sequences of these 11 MdCBLs were 187–259 amino acids (aa) in length, with predicted mass weight, isoelectric point (pI), and charge at pH 7.0 ranging from 21.38 to 29.53, 4.42 to 4.89, and –10.78 to –17.30, respectively (Table 1). 

**Figure 1:**
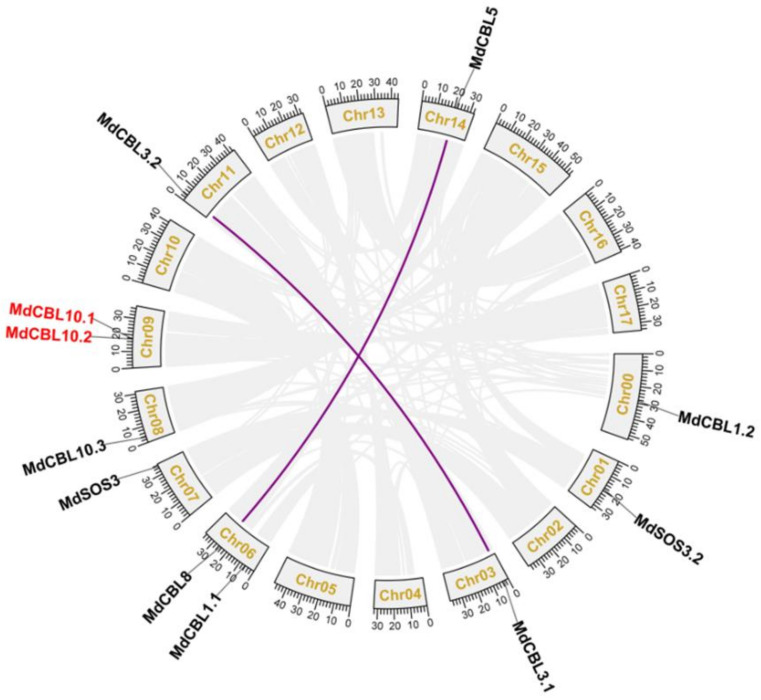
Genome locations of CBL family genes in apple. Purple lines and red font indicate the segmental and tandem duplication genes, respectively. Collinear blocks are represented by grayish lines. Chr00 represents the unassembled genomic scaffolds.

Based on the genomic location information obtained from the GDR database, 10 of the 11 *MdCBL* genes were randomly distributed on 8 of the 17 chromosomes of the apple, and the remaining one (*MdCBL1.2*) was localized to unassembled genomic scaffolds (Table 1 and Figure 1). Segmental and tandem duplications are the main causes of gene family expansion in plants. The collinear analyses between different apple chromosomes were performed with MCScanX software to investigate the gene duplication events among these *MdCBL* genes. The results showed complex patterns of collinearity between different chromosomes. The collinear analysis also revealed two segmental duplication events (*MdCBL3.1* and *MdCBL3.2*, and *MdCBL5* and *MdCBL8*) and one tandem duplication event (*MdCBL10.1* and *MdCBL10.2*) among these *MdCBL* genes (Figure 1).

### 3.2. Phylogenetic Analysis, Gene Structure Display, Prediction of Conserved Motifs, and Cloning of Apple CBL Family Genes

**Figure 2:**
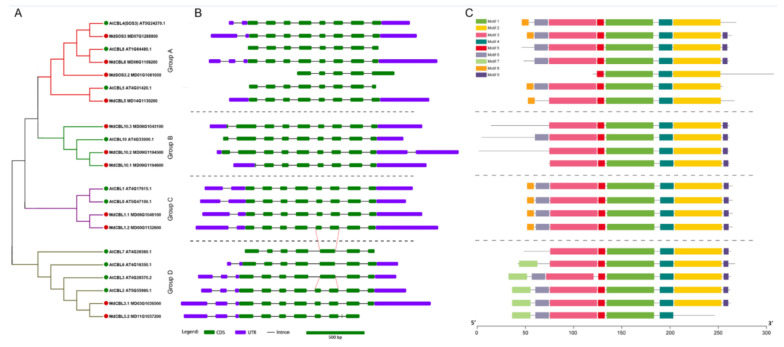
Phylogenic relationships (**A**), gene structure (**B**), and conserved motifs (**C**) for CBL family proteins in apples and *Arabidopsis*. Green and red dots indicate CBL proteins in *Arabidopsis* and apple, respectively. Red lines show that the fifth exon of *AtCBL7* and *AtCBL3* could be further differentiated into two shorter exons. Introns are represented by black line segments of the same length to facilitate the comparison of the exon–intron composition patterns of these genes.

Previous studies showed that the members of the CBL family in plants could be divided into four groups. A phylogenetic analysis based on the protein sequences of the 11 MdCBLs and 10 AtCBLs was performed to investigate the relationship between these 11 CBL family members in apples. The phylogenetic tree showed that the 11 MdCBL proteins could be divided into four groups, similar to the grouping results in *Arabidopsis* and many other plant species (Figure 2A). 

Gene structure is one of the factors that reflects the evolution of a multigene family. Thus, the exon–intron composition patterns of these *MdCBL* and *AtCBL* genes were analyzed. Although the length of introns varied significantly (Figure S2), genes that belonged to the same group exhibited the same exon–intron composition patterns, with the largest variation in the N-terminal region of these genes (Figure 2B). Based on gene structure analysis, three *MdCBL* genes with partial exons missing compared with other *CBL* genes in the same group, namely *MdSOS3.2*, *Md10.1*, and *MdCBL3.2*, were also identified. This result suggested that the predicted coding sequences of these three genes in apple genome (GDDH13) might be wrong. The conserved motifs of the CBL family were further explored using the online software MEME, and nine conserved motifs were found (Figure S3). The motifs 1–5 were conserved in almost all CBL proteins except MdSOS3.2 and MdCBL3.2 (Figure 2C). Besides, MdCBL10.1 was the missing part of the N-terminal compared with other proteins in group B (Figure 2C). These results further supported the presence of errors in the prediction of coding sequences of these three genes.

Gene-specific primers were designed based on the predicted sequences in the apple genome (GDDH13) and used for gene cloning to confirm the coding sequence of *MdCBL* genes. Using total RNA extracted from the leaves of “Golden Delicious” apple as the template, the coding sequences of these *MdCBL* genes were finally obtained by polymerase chain reaction (PCR) amplification, except for *MdSOS3.2* (Supplementary File S2). The sequence alignment showed that the predicted protein sequences of MdCBL10.1 (MD09G1194600) and MdCBL3.2 (MD11G1037200) were missing a segment of N-terminal and C-terminal, respectively (Figure S4A). Although the coding sequence of *MdSOS3.2* was not obtained, the sequence comparison between MD01G1081000 and MdSOS3 indicated an incorrect fragment deletion and an incorrect fragment insertion in the N- and C-terminals of MD01G1081000, respectively (Figure S4B). 

### 3.3. Promoter Analysis of MdCBL Genes

**Figure 3:**
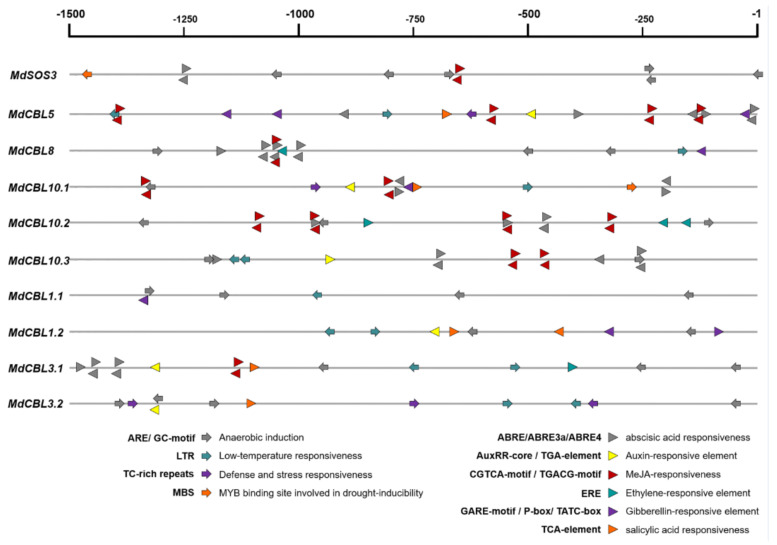
*Cis*-element analysis of the *MdCBL* gene promoter regions in apple. Arrows and triangles indicate the *cis*-elements related to abiotic stress response and hormone response, respectively. Positive and negative directions indicate whether the motif existed in the plus or minus strand of the *cis*-acting elements, respectively.

CBL family genes are involved in plant response to various environmental stresses. The promoter regions (upstream 1500 bp of the start codon ATG) of these genes were obtained from the apple genome (GDDH13) and submitted to the online software PlantCARE for *cis*-acting element analysis to explore the possible response of *MdCBL* genes to abiotic stresses. Various *cis*-elements related to abiotic stresses (hypoxia, cold, and drought) and plant hormone (ABA, auxin, MeJA, ethylene, GA, and SA) responsiveness were found (Figure 3 and Supplementary File S3), suggesting that these *MdCBL* genes played an important role in apple stress response. Many of these *cis*-elements appeared multiple times in the promoter region of the same gene (Figure 3). Besides, more hormone response–related *cis*-elements were present in the promoter regions of *MdCBLs* in groups A and B, especially *cis*-elements related to ABA and MeJA, while the *cis*-elements in the promoter regions of *MdCBLs* in groups C and D were mostly related to abiotic stress response (Figure 3). This indicated the functional differentiation of *MdCBL* genes between different groups.

### 3.4. Functional Identification of Mdcbls in Regulating Salt Tolerance in Yeast 

**Figure 4:**
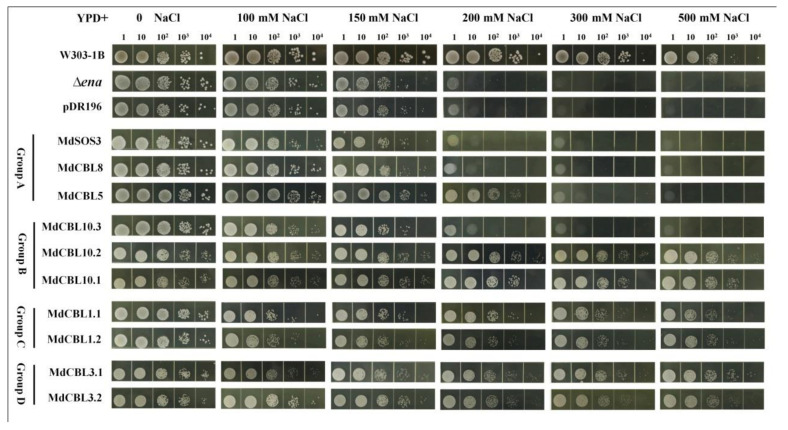
Functional identification of *MdCBLs* in the Na^+^-sensitive yeast mutant. Aliquots (10 µL) of serial dilutions (10^0^, 10^1^, 10^2^, 10^3^, and 10^4^) were dotted onto the YPD medium in the presence of 0, 100, 150, 200, 300, and 500 mM NaCl and grown for 3 days. W303-1B was the positive control. Δ*ena1−4* and Δ*ena1−4* transformed with the pDR196 empty vector were used as negative controls.

Previous studies demonstrated that CBL proteins affected plant salt tolerance through the SOS pathway. The yeast mutant strain *Δena*1−4 that lacked Na^+^-ATPase and was sensitive to high [Na^+^] was used to identify which CBL proteins in apples had a significant regulatory effect on salt tolerance. The full-length coding sequences of the ten *MdCBL* genes were cloned into the pDR196 vector and then transformed into the yeast mutant *Δena*1−4. The yeast strain W313-1B and positive transformants of the empty vector pDR196 were used as positive and negative controls. All of these strains were cultured in a YPD medium containing different concentrations of NaCl for three days. The growth of all strains was almost the same in a normal YPD medium (0 NaCl) (Figure 4). The addition of NaCl significantly inhibited the growth of these strains. The growth of the yeast mutant *Δena*1−4 was almost completely inhibited at a 200 mM NaCl concentration. The overexpression of any one of the seven *MdCBL* genes (MdCBL5, MdCBL10.1, MdCBL10.2, MdCBL1.1, MdCBL1.2, MdCBL3.1, and MdCBL3.2) could significantly inhibit the sensitivity of *Δena*1−4 to a high NaCl concentration (Figure 4). These results indicated that these seven *MdCBL* genes played a positive role in modulating salt tolerance in yeast. The growth of *MdCBL5* transgenic yeast was also completely inhibited with the further increase in the NaCl concentration (Figure 4), indicating a stronger capacity of the other six *MdCBL* genes than *MdCBL5* in enhancing yeast salt tolerance.

### 3.5. Overexpression of MdCBL10.1 Improved Salt Tolerance of Cisgenic Apple Calli

The SOS3/CBL10-SOS2-SOS1 and CBL10-CIPK8-SOS1 signaling pathways are the paramount regulatory mechanisms for facilitating Na^+^ extrusion and are critical to the ability of plants to adapt to high salinity [31,41]. Based on the functional identification results in yeast, *MdCBL10.1* was selected for the subsequent transgene and functional identification in apple. Full-length CDS of *MdCBL10.1* was cloned into the pBI121 vector and transformed into apple calli. PCR identification and quantitative reverse transcription (qRT)-PCR expression analysis demonstrated that several cisgenic lines with high *MdCBL10.1* expression were obtained (Figure S5). Three lines (OE-3, OE-4, and OE-7) with high *MdCBL10.1* expression levels were selected for NaCl treatment. 

**Figure 5:**
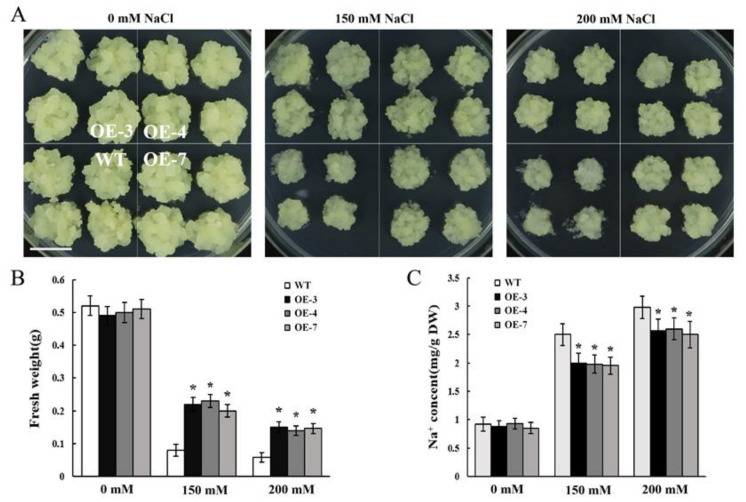
Functional identification of *MdCBL10.1* in cisgenic apple calli. (**A**) Phenotypes of the cisgenic (OE) and wild-type (WT) apple calli treated with NaCl stress. The scale bar represents 1 cm. Fresh weight (**B**) and Na^+^ content (**C**) in cisgenic and WT apple calli. For (**B**) and (**C**), error bars represent the SD of three independent biological replicates, with each biological repeat having at least three dishes. Bars labeled with * in each panel are significantly different from the WT (*p* < 0.05, Student *t* test).

After 20 days of culture, no significant difference was found between OE lines and wild type (WT) on the normal MS medium. The growth of all lines was inhibited under NaCl treatment, with significantly reduced fresh weight compared with the apple calli cultured in MS medium (Figure 5A,B). However, the growth of *MdCBL10.1* cisgenic lines was significantly better, and the fresh weight was significantly higher than that of the WT under salt treatment. These results suggested that the overexpression of *MdCBL10.1* could significantly improve the salt tolerance of cisgenic apple calli (Figure 5A,B). In *Arabidopsis*, CBL10 participates in the SOS pathway and promotes the Na^+^ efflux [31,41]. Therefore, the Na^+^ content of the apple calli was measured. No significant difference was observed between different lines cultured in the normal MS medium. Under NaCl treatment, the Na^+^ contents of three cisgenic lines were significantly lower than that of the WT (Figure 5C), suggesting that the overexpression of *MdCBL10.1* could inhibit the excessive accumulation of Na^+^ in cisgenic apple calli. 

### 3.6. Overexpression of MdCBL10.1 in Roots Enhanced the Salt Tolerance of Apple Plants

The use of *A. rhizogenes* K599 to obtain plants with cisgenic roots provides a convenient way for studying the function of *MdCBL10.1* in apple plants because of the low genetic transformation efficiency [39,40]. Full-length CDS of *MdCBL10.1* was cloned into the pCAMBIA2300 vector fused with a GFP tag, and a selected inhibitory fragment of *MdCBL10.1* was cloned into the RNA-interference vector pK7GWIWG2D. Through GFP fluorescence detection and expression analysis (Figure S6), 20 plants with high *MdCBL10.1* expression (MdCBL10.1-OE) and 20 plants with *MdCBL10.1* expression significantly inhibited (MdCBL10.1-RNAi) were selected for subsequent salt treatment. Plants with their roots transformed with empty vectors pCAMBIA2300-GFP (OE-EV) and pK7GWIWG2D (RNAi-EV) were used as controls. 

**Figure 6:**
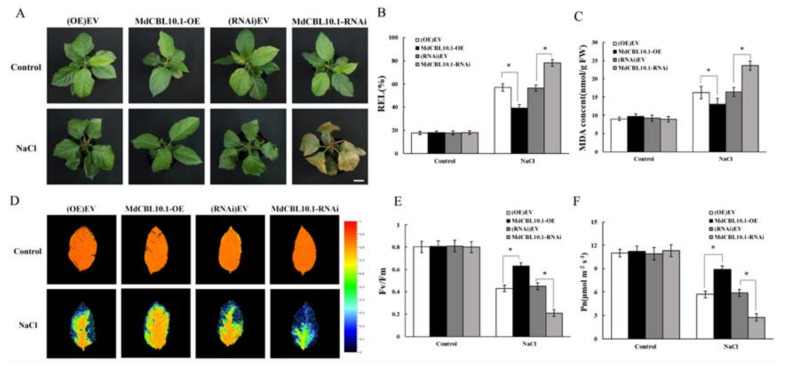
Phenotypic analysis of *MdCBL10.1* cisgenic apple plants under 150 mM NaCl treatment. (**A**) Growth phenotypes of cisgenic apple plants after NaCl treatment. Scale bars represent 3 cm. (**B**) Relative electrolyte leakage (REL) of apple leaves. (**C**) Malondialdehyde (MDA) content in apple leaves. Representative chlorophyll fluorescence images (**D**), Fv/Fm ratios (**E**), and net photosynthetic rate (Pn) (**F**) of cisgenic apple plants under normal and NaCl stress conditions. (OE)-EV and (RNAi)-EV represent roots transformed with the empty vector pCambia2300 and pK7GWIWG2D, respectively. The values of each index for cisgenic plants of the same type are the average values from all lines. Values are means of 20 replicates ± SD (each plant acts as a biological replicate). * in each panel denotes values significantly different from the corresponding control lines (*p* < 0.05, Student *t* test).

Under normal conditions (control group), no significant difference was observed between different types of cisgenic plants. After ten days of 150 mM NaCl irrigation, the RNAi plants were severely damaged by salt treatment, with their leaves showing obvious yellowing, wilting, and even death. Compared with the RNAi and control lines, the OE plants exhibited a better growth state, with their leaves still bright green and vigorous (Figure 6A). The measurements of relative ion leakage (REL) and MDA content in the leaves of these plants also indicated that the OE plants suffered less, whereas the RNAi plants suffered more stress damage caused by NaCl treatment (Figure 6B,C). 

Besides REL and MDA content, the maximum quantum yield of PSII (Fv/Fm) is also an appropriate indicator for the early identification of the degree of damage in plants. Under normal conditions, the leaves of all lines were healthy and maintained high Fv/Fm ratios (Figure 6D,E). After NaCl treatment, the Fv/Fm of leaves of the RNAi plants decreased significantly, while that of the OE plants remained high (Figure 6D,E). The damage to photosynthetic units directly affected photosynthesis. After NaCl treatment, the net photosynthetic rate (Pn) of RNAi plants was significantly lower, while that of the OE plants was significantly higher than that of the control lines (Figure 6F). This was consistent with the performance of Fv/Fm and further supported that the overexpression of *MdCBL10.1* alleviated the stress damage to apple plants caused by NaCl treatment.

**Figure 7:**
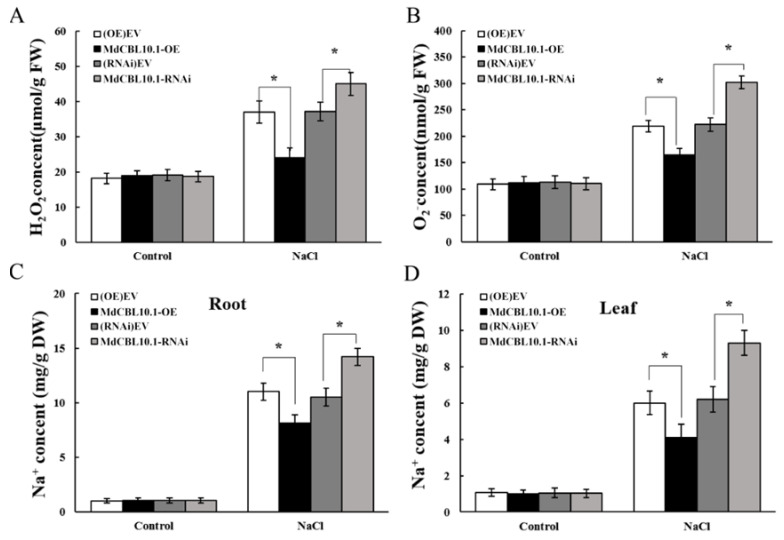
Overexpression of *MdCBL10.1* in apple roots inhibited the ROS and Na^+^ accumulation in cisgenic apple plants. (**A**) Hydrogen peroxide (H_2_O_2_) content in roots. (**B**) Superoxide anion (O_2_^−^) content in roots. (**C**) Na^+^ content in roots. (**D**) Na^+^ content in leaves. The values of each index are the average values of all lines in cisgenic plants of the same type. Values are means of 20 replicates ± SD. * in each panel denotes values significantly different from the corresponding control lines (*p* < 0.05, Student *t* test).

Salt stress triggers the accumulation of reactive oxygen species (ROS), which is harmful to plant growth. The accumulation of ROS in the roots of these NaCl-treated apple plants was determined to evaluate the damage caused by salt stress to root systems. The results showed that the roots of the OE lines accumulated less H_2_O_2_ and O_2_^−^, while the roots of the RNAi plants accumulated more ROS than controls (Figure 7A,B). Since *MdCBL10.1* overexpression could inhibit Na^+^ accumulation in apple calli, the Na^+^ content in these apple plants after NaCl treatment was measured. As expected, the Na^+^ content in the roots and leaves of OE plants was significantly lower than that of the controls, while the RNAi plants showed the opposite trend (Figure 7C,D). These results indicated that *MdCBL10.1* overexpression in roots could reduce the Na^+^ content in both roots and leaves, thereby alleviating salt stress-induced damage to apple plants. 

## 4. Discussion

Plants are inevitably exposed to a variety of adverse environmental conditions, such as water shortage, low temperature, high salinity, and so forth, due to the sessile lifestyle. These abiotic stresses are increasingly harmful to crop yield and quality. Calcium and its signaling pathway play an important role in plant stress response. As specific Ca^2+^ sensors, CBL proteins play vital roles in calcium signaling and stress resistance regulation [7]. Apple is one of the most economically important fruits in the world. Its cultivation and extension are restricted by various abiotic stresses. The study of CBL proteins is thus important for the resistance breeding of apple. To date, the CBL family proteins in many plant species have been identified at the genome-wide level. However, no detailed characterization of apple CBL family proteins has been reported, and little is known about their functions in abiotic stress response. 

Ca^2+^ is an essential element for plant growth and survival, and Ca^2+^ signals are an important regulator of growth, development, and biotic and abiotic stresses in plants [7,38,42]. Studies in various fruit tree crops also have shown that Ca^2+^ plays an important role in regulating fruit development, ripening, quality, and storage [43,44,45,46,47]. The CBL family has been identified and systematically studied in many plant species, such as dicotyledons *Arabidopsis* (10 AtCBLs) [33,48], eggplant (5 SmCBLs) [36], cotton (13 GaCBLs, 13 GrCBLs, 22 GhCBLs) [1], Cassava (9 MeCBLs) [12], pepper (9 CaCBLs) [10], tea plant (7 CsCBLs) [32], and pigeon pea (9 CcCBLs) [49], as well as the monocotyledon rice (10 OsCBLs) [33,48], due to the important role of CBL proteins in Ca^2+^ signaling. The CBL family has also been identified in some fruit trees such as grapevine (8 VvCBLs) [8], banana (11 MaCBLs) [50], and pineapple (8 AcCBLs) [11]. Although the genomes of these species are significantly larger than that of *Arabidopsis*, the number of *CBL* genes in most of these species is not significantly higher than that in *Arabidopsis* [48]. In this study, 11 *MdCBL* genes were identified from apples (Table 1), only one more than that from *Arabidopsis*, suggesting that the CBL family in apples had not expanded significantly during evolution. This was different from other gene families that were previously identified in apple, such as the bHLH [37], Lhc [51], and CaCA [38] families. Tandem and segmental duplication events are fundamental mechanisms of gene family expansion. Considering the two genome-wide duplication events that occurred during apple evolution [52], collinear analysis between these *MdCBLs* was performed. The results showed that only two segmental duplication events occurred (Figure 1), indicating that the CBL family did not greatly expand during apple evolution. The evolutionary analysis of many plant species also suggested that the number of CBL members was independent of their genome size [48].

Although the number of CBL members varied, the phylogenetic analysis of CBL proteins in various plant species indicated that this family should be divided into four groups. In this study, the MdCBL proteins were divided into four groups (groups A to D), as in *Arabidopsis* (Figure 2A). This grouping result was also supported by the gene structure analysis and conserved motif prediction. The intron/exon and motif composition patterns of genes within the same group were consistent, whereas significant differences were observed between different groups (Figure 2B,C). In addition, based on the comparison of gene structure and conserved motifs of genes within the same group, three *MdCBL* genes (*MdSOS3.2*, *MdCBL10.1*, and *MdCBL3.2*) that might have errors in their predicted CDS in the apple genome were identified. The gene cloning results confirmed this speculation (Table 1 and Figure S4) and further suggested that this comparative analysis method could help people identify genes in a gene family whose coding sequences were incorrectly predicted. On the contrary, the high similarity of gene structure, conserved motifs, and *cis*-acting elements (Figure 2 and Figure 3) suggested functional redundancy among *MdCBL* genes, especially *MdCBL* genes in the same group. Similar results were also found in *Arabidopsis*, such as SOS3 and CBL10 [31].

Stress stimulation causes a transient increase in the intracellular Ca^2+^ concentration. CBL proteins can sense and interact with intracellular increased Ca^2+^. The binding of Ca^2+^ promotes the interaction between CBL and CIPK proteins, which is crucial for the activation of the kinase activity of CIPKs. Then, the activated CIPKs phosphorylate downstream substrates, further triggering a range of response mechanisms [7,33,42]. Decades of research have revealed extensive and complex interaction networks between CBL and CIPK family proteins. As Ca^2+^ sensors, each CBL has three or more EF-hand domains and Ca^2+^-binding sites. Besides, the CBL proteins harbor a conserved FPSF domain in their C-terminal, which is the target of phosphorylation by CIPK. Moreover, many of the CBL proteins also contain conserved MGCXXS/T motifs in their N-terminal, which contribute to the anchorage of CBLs in the membrane to transduce a Ca^2+^ signal [48,49,50,53]. Several studies proved that different CBLs could be localized to the plasma or vacuole membrane and could regulate plant salt tolerance by promoting the Na^+^ efflux or sequestration [7,42]. In this study, all MdCBL proteins contained the FPSF domain in their C-terminal (Figure 2, Figures S3 and S4), suggesting complex interactions between MdCBLs and CIPK family proteins in apple. For example, four MdCBL proteins were found to interact with the CIPK family protein MdSOS2L1 [54]. Many of these MdCBLs contained the conserved MGCXXS/T domain in their N-terminal. Further, the distribution of this domain showed obvious group specificity, which was found only in groups A and C of the MdCBL family (Figure 2, Figures S3 and S4). These results suggested that MdCBL proteins in these two groups might be more likely to function on the membrane and also indicated the functional and subcellular differentiation of CBL proteins between different groups. More studies on the subcellular localization and functional identification of apple CBL proteins are needed to confirm this hypothesis.

The CBL-CIPK model is reported to characterize many forms of abiotic stress responses. The first identified CBL-CIPK pathway was the SOS pathway, which plays vital roles in plant salt tolerance regulation [55]. It contains three key components: SOS1 (Na^+^/H^+^ antiporter), SOS2 (CIPK24), and SOS3 (CBL4). Under salt stress, the SOS2–SOS3 complex activates the transport properties of SOS1 to promote the Na^+^ efflux, thus enhancing plant salt tolerance. Subsequent studies found that CBL10 (SCABP8) could also interact with SOS2 and activate SOS1, suggesting the functional redundancy between SOS3 and CBL10 [31]. Studies in apples also showed that MdCBL10 could interact with MdSOS2L1, an SOS2-like protein that played positive roles in regulating plant salt tolerance [54]. In addition, a recent study found that the CBL10–CIPK8 complex regulated plant salt tolerance through its interaction with SOS1 [41]. These results, combined with the functional identification results in yeast (Figure 4), led to the selection of *MdCBL10.1* for further genetic transformation and functional identification in apple. The phenotypic comparison and measurement of stress-related physiological indicators showed that *MdCBL10.1* overexpression significantly enhanced the salt tolerance of cisgenic apple materials (Figure 5, Figure 6 and Figure 7). Moreover, the significantly reduced Na^+^ content in transgenic and cisgenic materials indicated that *MdCBL10.1* overexpression could significantly inhibit Na^+^ accumulation under salt stress. These results suggested that MdCBL10.1 enhanced plant salt tolerance by inhibiting Na^+^ accumulation, and this was probably achieved through the SOS pathway.

In conclusion, 11 *MdCBL* genes were identified from apple. CDSs of these genes were cloned, and the gene structure and conserved motifs were analyzed. Several *MdCBLs* that played positive roles in salt tolerance were identified using the Na^+^-sensitive yeast mutant. The function of *MdCBL10.1* in regulating salt tolerance was also identified in cisgenic apple materials in detail. This study provided a foundation for future research examining the function and mechanism of CBL proteins in regulating apple salt tolerance.

## Figures and Tables

**Table 1:** Characterization of the CBL family genes in apple.

Group	Gene Name	Gene Locus (GDDH13)	Genomic Location (GDDH13)	Deduced Polypeptide				Best Hits
				Length (aa)	Mass Weight (kDa)	pI	Charge at PH 7.0	
**A**	MdSOS3	MD07G1288800	Chr07: 34980079–34983632	212	24.39	4.52	−16.44	AT5G24270
	**MdSOS3.2**	MD01G1081000	Chr01: 18815406–18816403	**187**	**21.38**	**4.53**	**−9.88**	AT5G24270
	MdCBL5	MD14G1130200	Chr14: 20784655–20786608	214	24.53	4.42	−15.87	AT4G26570
	MdCBL8	MD06G1109200	Chr06: 24877949–24881781	212	24.42	4.60	−13.48	AT1G64480
**B**	**MdCBL10.1**	MD09G1194600	Chr09: 17493583–17495782	**187 (266)**	**21.57 (30.51)**	**4.48 (4.88)**	**−14.55 (−11.27)**	AT4G33000
	**MdCBL10.2**	MD09G1194500	Chr09: 17485706–17488584	**259 (245)**	**29.53 (27.88)**	**4.54 (4.63)**	**−17.30 (−14.31)**	AT4G33000
	MdCBL10.3	MD08G1043100	Chr08: 3254626–3256803	246	28.28	4.65	−17.03	AT4G33000
**C**	MdCBL1.1*	MD06G1046100	Chr06: 6240265–6244670	213	24.52	4.63	−10.78	AT4G17615
	MdCBL1.2	MD00G1132600	Chr00: 28662543–28667386	213	24.52	4.63	−10.78	AT4G17615
**D**	MdCBL3.1	MD03G1036500	Chr03: 2901761–2905532	226	26.06	4.69	−14.17	AT4G26570
	**MdCBL3.2**	MD11G1037200	Chr11: 3199328–3202169	**210 (226)**	**24.20 (25.96)**	**4.89 (4.75)**	**−10.34 (−13.17)**	AT4G26570

Best hits in *Arabidopsis* were determined by local blastp method. Sequence length, mass weight, pI, and charge at PH 7.0 of the revised proteins (MdCBL10.1, MdCBL10.2, and MdCBL3.2) were listed in parentheses.

## Data Availability

All data supporting the findings of this study are available within the paper and within its supplementary data published online.

## Supplementary Materials

The following are available online at https://www.mdpi.com/article/10.3390/ijms222212430/s1. Figure S1: Phylogenetic analysis of proteins in HMMER screening results and CBL family members in Arabidopsis; Figure S2: Comparison of gene structure of the CBL family genes in apples and Arabidopsis; Figure S3: Putative conserved motifs identified in the sequences of apple CBL family proteins. Red dots indicate the conserved “FPSF” and “MGCxxS/T” domains; Figure S4: Sequence comparison between the predicted MdCBLs in the apple genome and the MdCBLs that were actually cloned in this study; Figure S5: Identification of cisgenic apple calli. (**A**) PCR identification of the MdCBL10.1 transgene based on genomic DNA extracted from apple calli. (**B**) Expression level of MdCBL10.1 in cisgenic apple calli. MdMDH served as an internal reference gene. Bars labeled with different letters in each panel are significantly different (*p* < 0.05, one-way ANOVA and Duncan’s test); Figure S6: Identification of the MdCBL10.1 transgene in the roots of apple plants. (**A**) GFP expression in cisgenic apple roots. (**B**) qRT-PCR determination of MdCBL10.1 expression in cisgenic apple roots. (OE)-EV and (RNAi)-EV represent roots transformed with the empty vector pCambia2300 and pK7GWIWG2D, respectively. MdMDH served as an internal reference gene. Bars labeled with different letters in each panel are significantly different (*p* < 0.05, one-way ANOVA and Duncan’s test). Supplementary File S1: HMMER screening results. Supplementary File S2: CDS and protein sequences of the cloned MdCBL genes. Supplementary File S3: Cis-elements identified in the promoter regions of MdCBL genes. Plus and minus signs indicate whether the cis-element was located in the plus or minus strand.
