# Supplementary figures and images for: Genome-Wide Analysis of the Apple CBL Family Reveals That Mdcbl10.1 Functions Positively in Modulating Apple Salt Tolerance
Source: Int J Mol Sci. 2021 Nov 18;22(22):12430. doi: 10.3390/ijms222212430 (PMC8624107; doi:10.3390/ijms222212430)

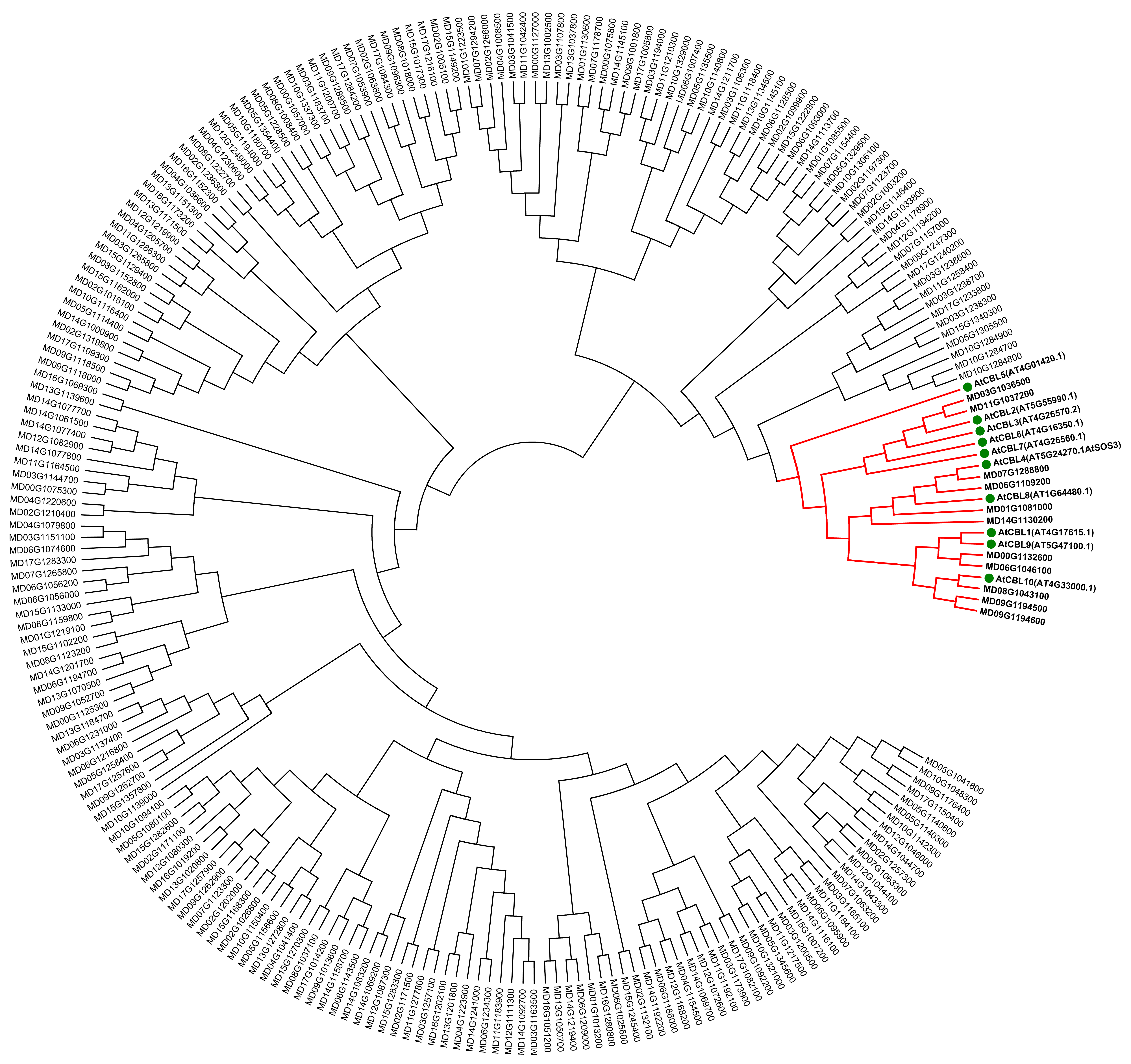

Supplement: Supplementary file 1 [file ijms-22-12430-s001.zip › Supplementary_Figure S1.jpg]

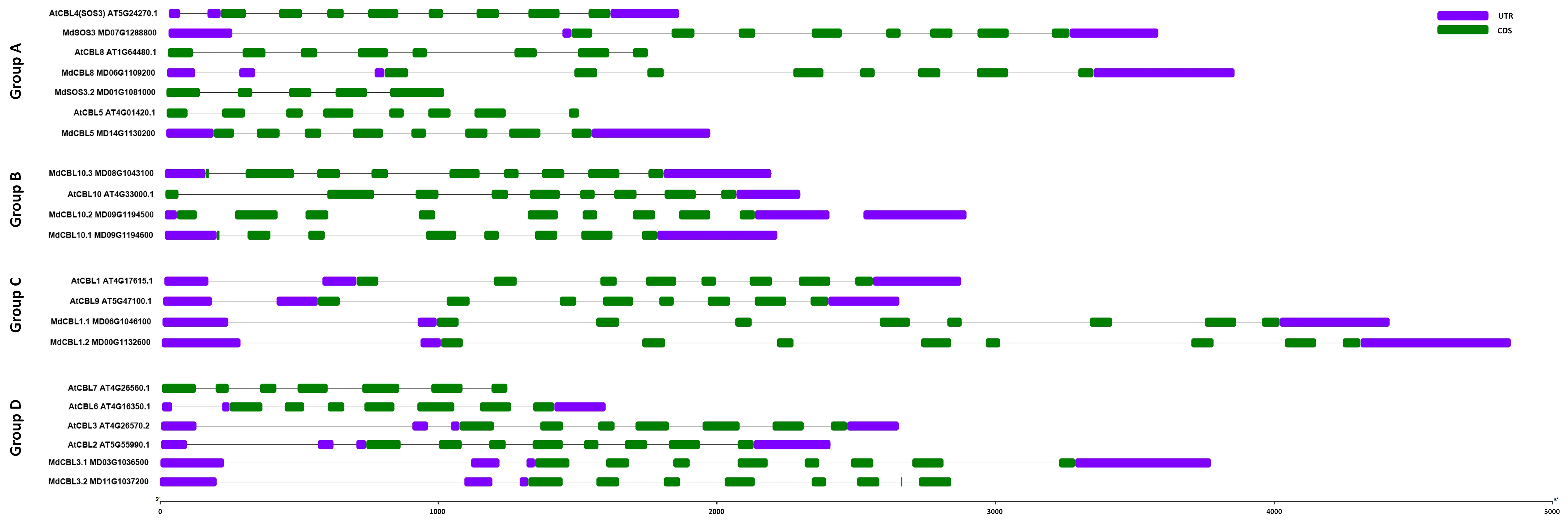

Supplement: Supplementary file 1 [file ijms-22-12430-s001.zip › Supplementary_Figure S2.jpg]

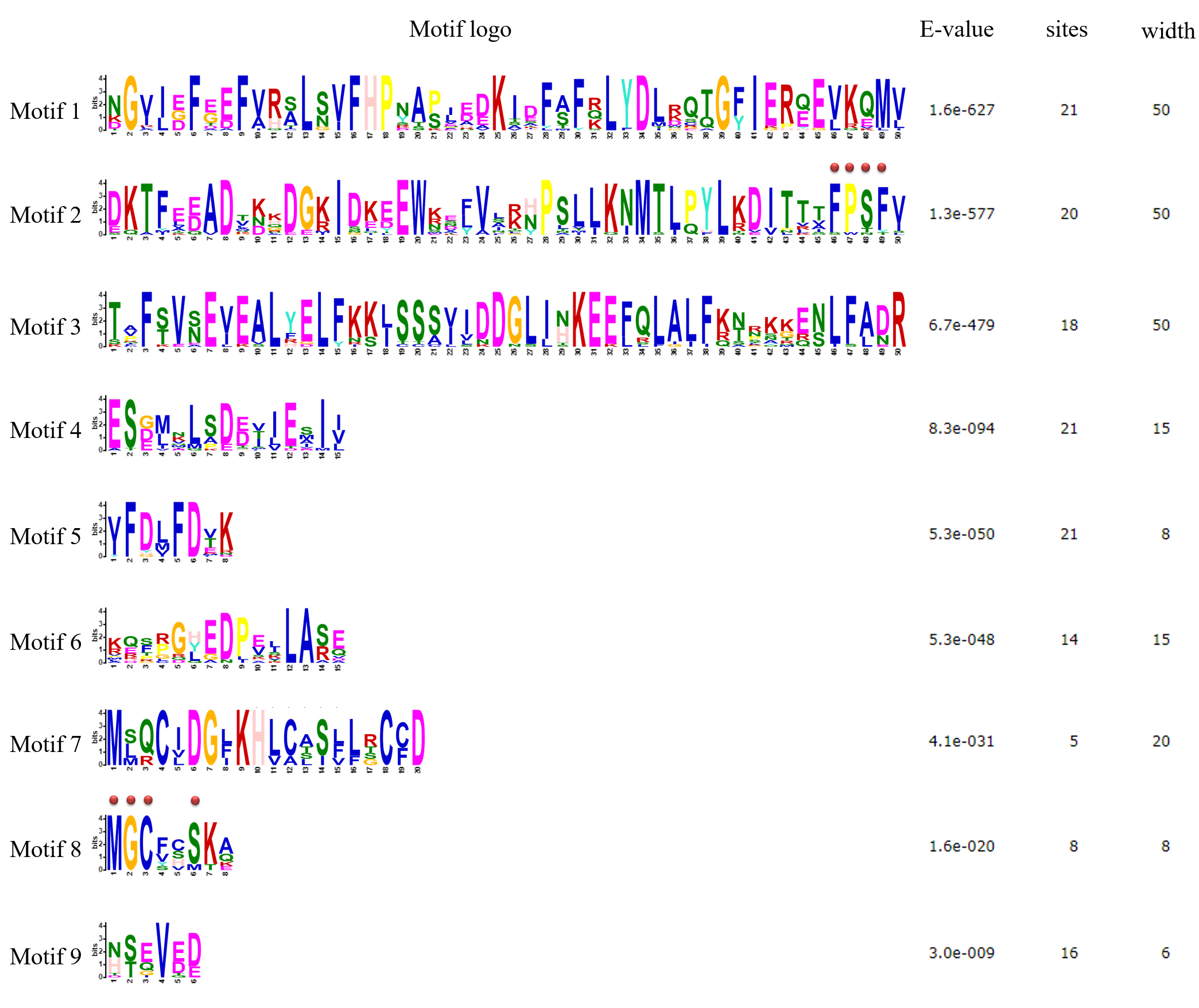

Supplement: Supplementary file 1 [file ijms-22-12430-s001.zip › Supplementary_Figure S3.jpg]

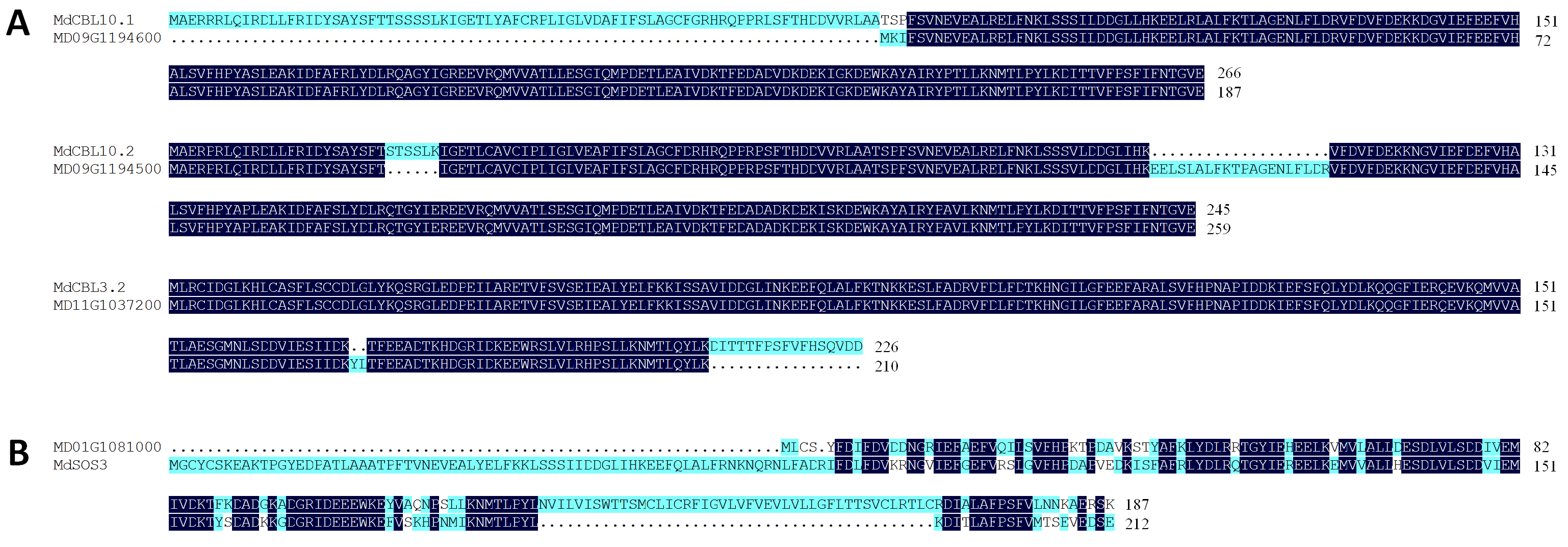

Supplement: Supplementary file 1 [file ijms-22-12430-s001.zip › Supplementary_Figure S4.jpg]

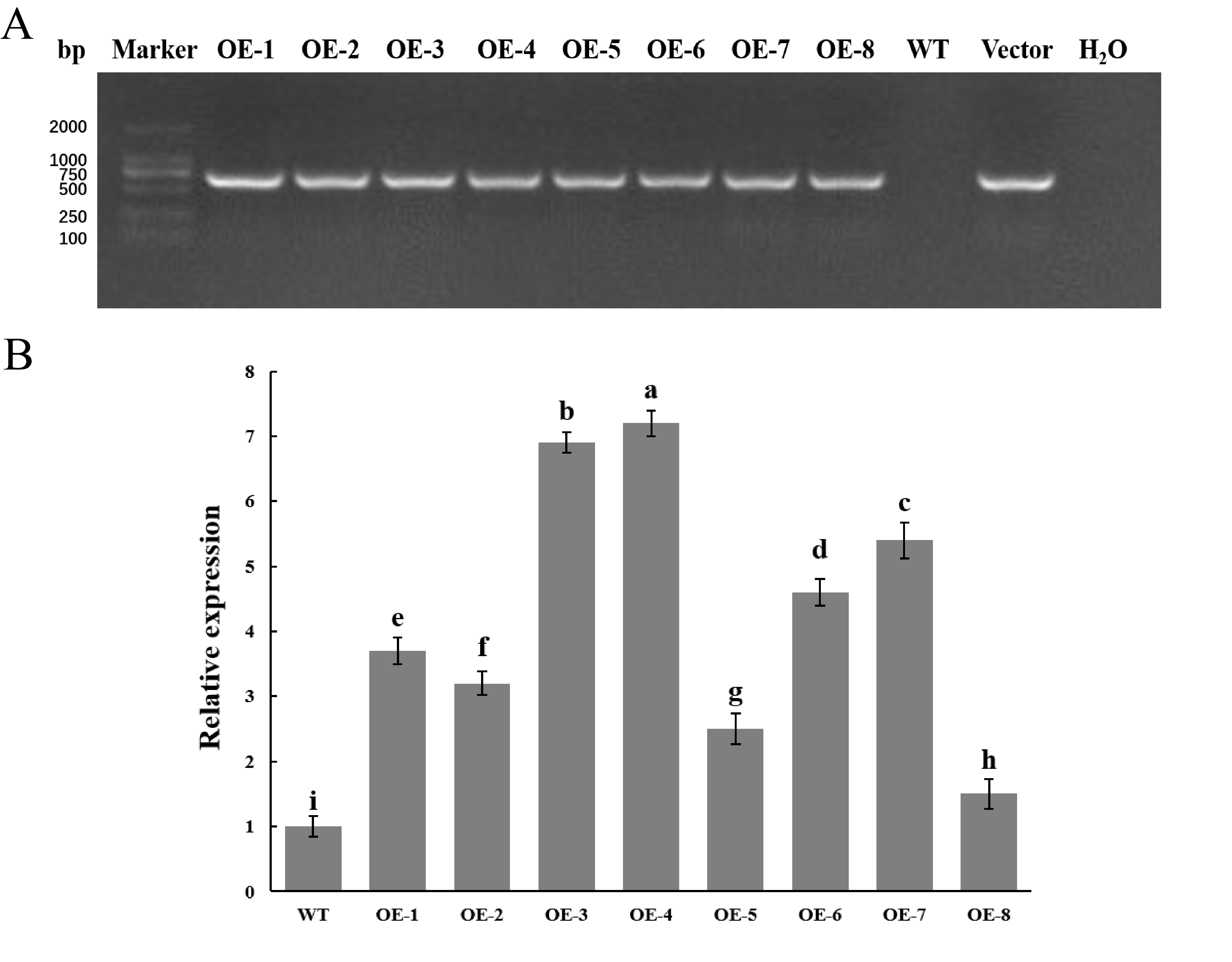

Supplement: Supplementary file 1 [file ijms-22-12430-s001.zip › Supplementary_Figure S5.jpg]

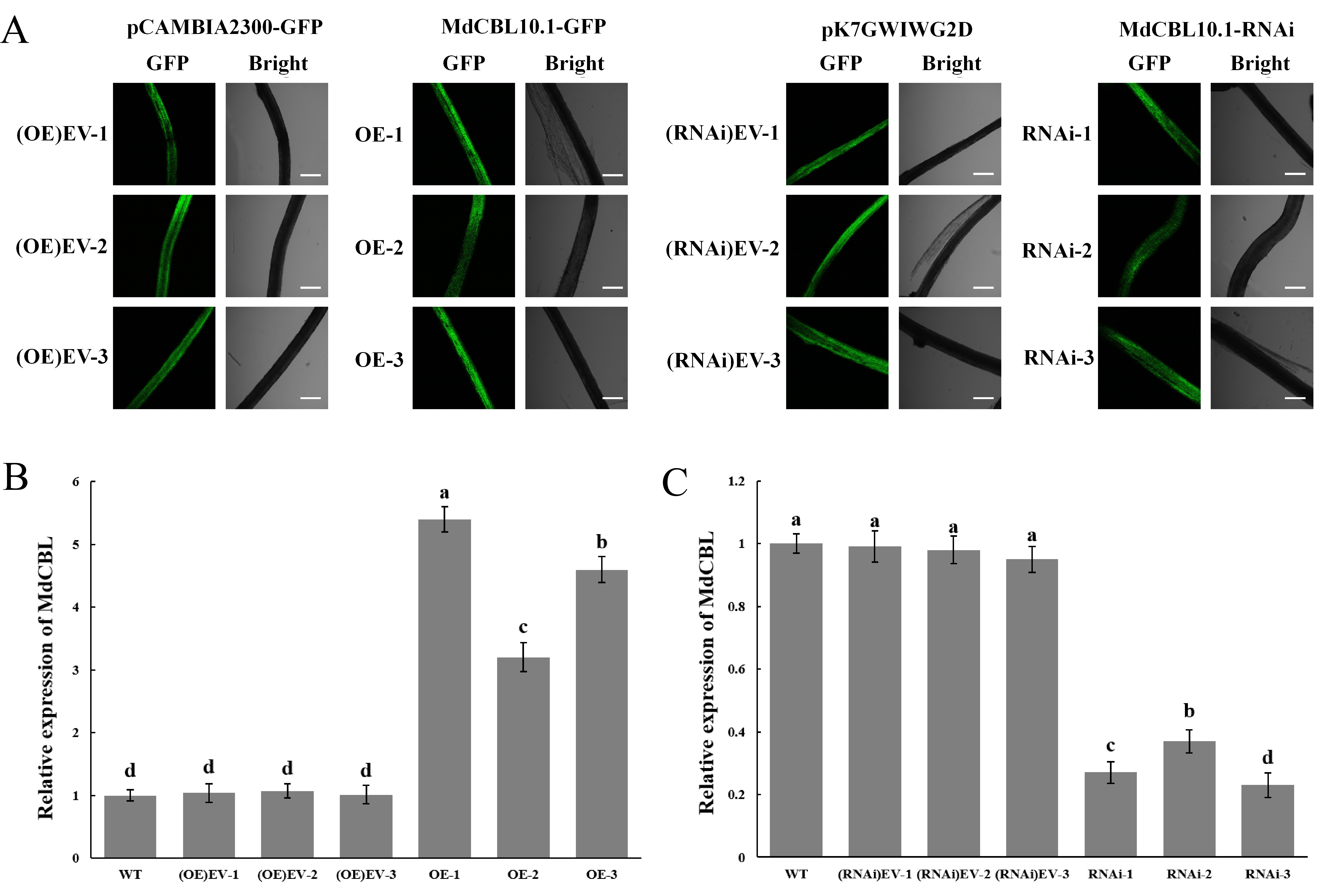

Supplement: Supplementary file 1 [file ijms-22-12430-s001.zip › Supplementary_Figure S6.jpg]
